# Behavioral, not self-reported, experiential avoidance predicts early treatment discontinuation at residential substance use treatment

**DOI:** 10.3389/fpsyt.2026.1725326

**Published:** 2026-02-06

**Authors:** Wenyue Wang, Dennis E. McChargue, Bilal Khan, Andrew P. Oakland

**Affiliations:** 1 Department of Psychology, University of Nebraska-Lincoln, Lincoln, NE, United States; 2 Department of Biostatistics & Health Data Science, College of Health, Lehigh University, Bethlehem, PA, United States

**Keywords:** alcohol use, emotion regulation, experimental paradigm, survival analysis, treatment retention

## Abstract

**Objectives:**

A large proportion of unhoused individuals in residential substance use treatment discontinue treatment, though a longer stay in treatment consistently predicts successful recovery. Experiential avoidance (EA) as an important function underlying dysregulated emotions may contribute to negative treatment outcomes. Limited evidence is available for the effect of EA in predicting treatment discontinuation. The current study aimed to investigate the roles of both behavioral and self-reported EA in predicting early treatment discontinuation among unhoused individuals receiving residential substance use treatment.

**Method:**

Fifty-five male participants were recruited from a residential treatment facility. Upon admission, participants rated their EA using the Acceptance and Action Questionnaire (AAQ) and completed a word-based Algebra Avoidance Task, with EA being indexed by premature termination of the task. Participants’ treatment completion status and days spent in treatment were obtained upon discharge. Cox proportional hazards regression analyses were conducted to predict time until treatment discontinuation, controlling for age, years of education, and psychological distress.

**Results:**

Results suggest a significant effect of behavioral, but not self-reported, EA in predicting early treatment discontinuation, such that greater behavioral EA was associated with an increased hazard of early treatment discontinuation, *HR* = 2.50, 95% CI [1.09, 5.73], *p* = .030. Participants with a primary alcohol (versus non-alcohol substance) use disorder diagnosis were less likely to have demonstrated behavioral EA.

**Conclusions:**

Behavioral measures might provide a more proximal measure of EA than self-report. Treating EA may help improve treatment retention. More research is needed to extend the current findings and clarify the mechanisms.

## 1 Introduction

Individuals with substance use disorders (SUDs) experience significant health risks, yet only 19.3% of affected individuals in the US receive treatment (1). Among individuals receiving long-term (i.e., more than 30 days) residential SUD treatments, barriers to accessing and completing treatment are substantial (2), with only approximately half of residents being able to complete their planned treatment (3). Treatment completion rates were even lower among unhoused residents (20% for individuals aged 12–17 and 41.7% for those aged 18 or older) (3). Reasons for discontinuing treatment early (i.e., before clinically recommended completion) may include leaving against professional advice, incarceration, termination by facility, and transfer to another facility (4). Retention is critical, as a longer treatment stay and completion are linked to better outcomes (5, 6).

Emotion dysregulation contributes to negative consequences associated with SUDs, particularly related to heightened negative emotions and regulation struggles (7, 8). A key factor is experiential avoidance (EA), a tendency to avoid contact with distressing thoughts, emotions, and sensations and to take actions to alter these events and related contexts (9, 10). Specifically, individuals may engage in substance use behaviors as a short-term coping strategy to alter a wide range of negative experiences including boredom, anxiety, withdrawal sensations, which may vary with the psychoactive effects of the specific substance (9). The acute effects of substances in alleviating the aversive results of excessive use may further serve as a method of EA to maintain substance use patterns (11). Higher EA was found to significantly predict stronger craving (12), excessive substance use (13, 14), and more negative treatment outcomes (15), making it a crucial target for intervention.

Avoidance of distress can further hinder therapy engagement and completion. Residential SUD treatment is a setting where clients receive intense, group-based care that targets more severe substance use difficulties (16), which creates unique challenges for clients. The stress experienced during residential treatments, if not appropriately coped with, may persist or even increase throughout the treatment episode, leading to negative events that are tied to early termination of services. Individuals with heightened EA, for example, may respond to various challenges during residential treatment by not showing up in therapy sessions, returning to substance use, or even terminating treatment prematurely, despite the potential long-term benefits of exposure to these stressors (e.g., unlearning triggers of substance use, building more adaptive coping skills).

There was limited research examining the association between EA and negative SUDs treatment outcomes with inconclusive findings. Among daily smokers participating in a smoking cessation trial, decreases in cognitive-based smoking-specific EA were significantly associated with higher rates of nicotine abstinence and less severe withdrawal and affect symptoms on quit day (17). Avoidant coping was a significant predictor of poorer residential alcohol use treatment outcomes after five years, though only among residents with low self-efficacy (15). In contrast, avoidant coping did not significantly predict length of stay among recipients of an outpatient SUD treatment (18). As far as we know, no previous study has directly investigated the effect of EA in predicting early treatment discontinuation. Furthermore, investigations on EA and SUD outcomes, like the three studies reviewed above (15, 17, 18), tended to use self-report rather than behavioral measures of EA. This field of research will benefit from inclusion of experimental measures which may be able to more fully capture the behavioral nuances of EA.

The current study thus aimed to examine the link between a behavioral index of EA and early discontinuation of residential SUD treatment among unhoused individuals. As there was no existing behavioral measure of EA, we operationalized EA in accordance with prior work demonstrating its conceptual overlap with distress tolerance (19–22), using early task termination as an index of behavioral avoidance (23). The primary question of the current study was whether behavioral EA significantly predicted early treatment discontinuation. As discussed above, a more comprehensive assessment of EA requires both self-report and experimental measures. The current study thus also examined the associations of self-reported EA with early treatment discontinuation. We hypothesized that greater behavioral and self-reported EA would result in a shorter time to treatment discontinuation. We further explored characteristics differentiating individuals who demonstrated behavioral EA from those who did not.

## 2 Methods

### 2.1 Sample identification and setting

Participants were recruited from a men’s residential substance use treatment facility in a Midwestern city. The treatment facility was a non-hospital, residential transitional living treatment program for individuals recovering from SUDs who functioned independently in most life areas. The clients were adult males in partial remission from SUDs who had gone through at least 30 days of detoxification in another facility. Treatments provided at the residential treatment program included case management (24) as well as individual and group therapies for SUDs and other mental health conditions based on the relapse prevention model (25) with components of 12-step facilitation (26). The standard length of treatment was six months. The facility did not provide any medication-assisted treatment (MAT). However, residents were able to obtain psychiatric medications and MAT from an external psychiatric provider during the study period.

The inclusion criteria of the present study were: i) a current DSM diagnosis of a severe SUD in partial remission, ii) currently unhoused and unemployed, and iii) not currently enrolled in school. According to the US Department of Housing and Urban Development (27), to qualify as unhoused, the participant had to lack a fixed, regular, and adequate nighttime residence, including: i) living in a primary nighttime residence, either public or private, that was not meant for human habitation, ii) living in a temporary public or private shelter, or iii) exiting an institution where they had resided for no longer than 90 days and immediately before entering that institution, having lived in an emergency shelter or place not meant for human habitation.

The exclusion criterion of the present study was either less than eight years of formal education or a bachelor’s degree or equivalent. This criterion was designed considering the academic, skill-based nature of the mathematics-based EA task, because individuals with limited educational experiences might experience unduly high levels of discomfort going through the task, while those with higher academic achievements possibly experience minimal levels of distress. The current study was not pre-registered. It was approved by the institutional review board of the University of Nebraska-Lincoln. Data were collected from January to October 2014.

### 2.2 Procedure

**Figure 1:**
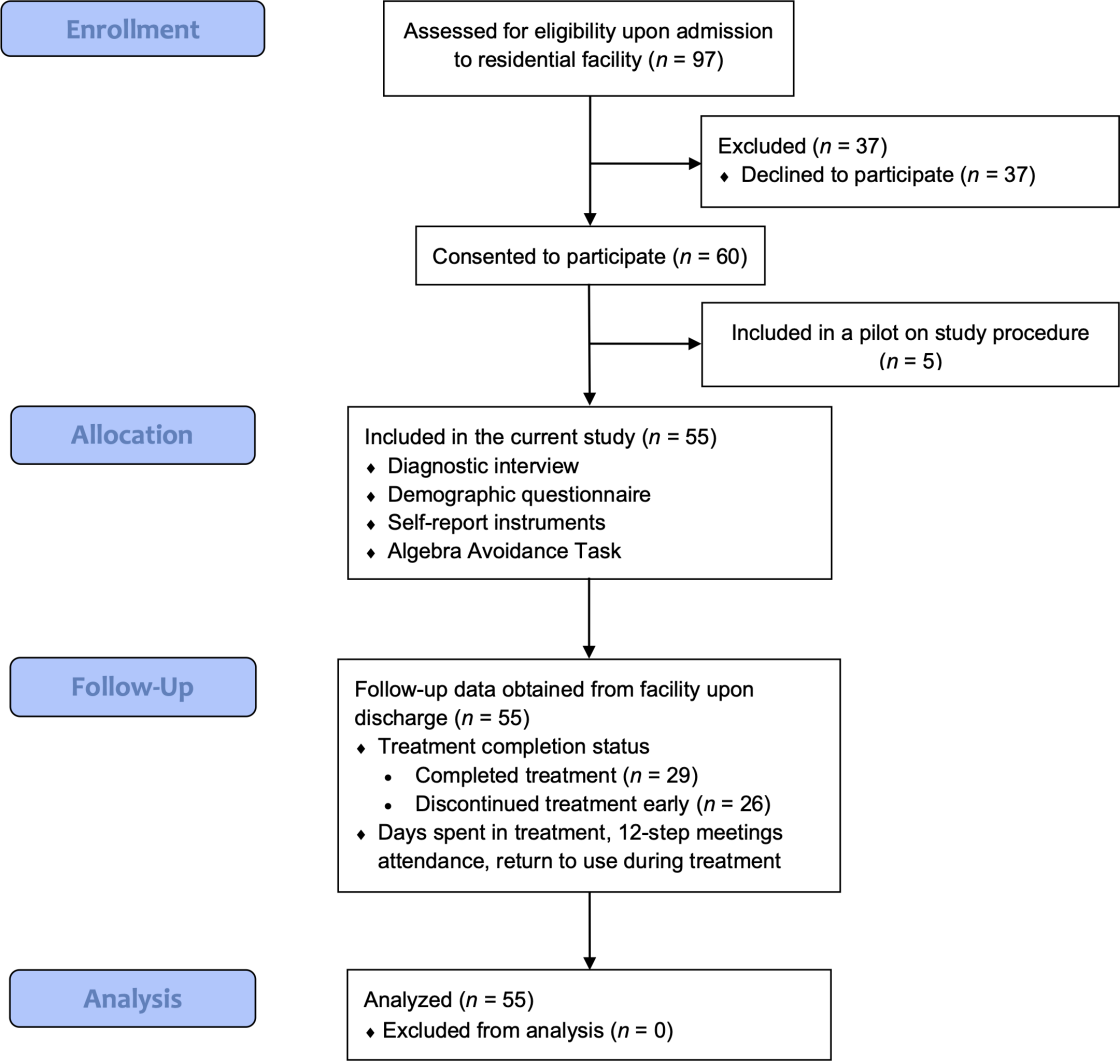
CONSORT flowchart.

**Figure 1** provides a CONSORT flowchart of study procedure. All individuals entering residential treatment at the facility during the nine-month study period were offered a chance to participate in the present study upon admission to the facility. Upon admission to the facility, all participants gave informed consent, went through a diagnostic interview by clinicians at the facility, and completed the intake assessment for the current study consisting of a demographic questionnaire, self-report instruments, and the Algebra Avoidance Task (AAT). The intake assessment took 30 to 45 minutes depending on whether the participant quit the AAT early.

Upon discharge from the facility, each participant’s treatment completion status (discontinued early versus completed), days spent in treatment, and other treatment-related information were obtained from the facility as part of the facility’s outcome monitoring. Whether an individual terminated treatment early during study period was determined by the treatment facility’s standards. The dates of admission and discharge varied across participants, though their intake and follow-up data were always collected at admission and upon discharge, respectively. Participants received a $3 remuneration for their participation and an additional amount depending on task performance (see details in section “Instruments”), which added to a total amount of up to $12.

### 2.3 Instruments

At intake, participants reported their age, gender, race/ethnicity, marital status, and years of education. Information about the primary and secondary SUD diagnoses, number of prior residential substance use treatment episodes, and medication status were recorded from the clinician-administered diagnostic interview. Participants also completed the 18-item Brief Symptom Inventory (BSI-18) (28), the Distress Tolerance Scale (DTS) (29), and the Acceptance and Action Questionnaire (AAQ) (10). The primary outcome of interest, time until early treatment discontinuation, was defined as the time (in days) until a participant either discontinued treatment before clinically recommended completion or was censored (i.e., early discontinuation not observed). Other treatment-related information collected included days spent in treatment, the number of 12-step meetings attended, and whether an individual returned to substance use during treatment.

The AAT was a stress-inducing behavioral task specifically developed for the current investigation. It was adapted from the mathematics tasks used in relevant studies (23, 30) and composed of nine word-based algebra problems of increasing complexity (**Supplementary Material 1**). Individuals were given a certain amount of time to complete each question and were instructed to move on to the next question whenever time had expired. To account for the increasing complexity of the questions, more time was allowed to work on later, more challenging questions (see **Supplementary Table 1** for detailed time allotments). The total time allowed was 20 minutes. Participants were given the full allotment of time regardless of their performance. They were instructed that they could quit the task at any time with no penalty and that they would not be given any remaining questions once they made the decision to quit. They were informed that they would be paid $3 for participation and an additional $1 for each test question answered correctly.

The task was designed in such a way (e.g., increasingly challenging problems, time constraints) that it was unlikely any participant would correctly answer all of them. Participants would therefore need to cope with the test-associated distress, like quitting the task early or persisting through it. To orient the participants to the types of mathematics questions being tested on, they were given two untimed practice questions prior to the beginning of the formal test and were given positive feedback on correctly answered questions. If a participant answered either practice question incorrectly, they would receive brief instructions on the correct solution before proceeding to the first test question.

EA was coded as a dichotomous variable of whether an individual quit the task prematurely (behavioral EA) or answered all test questions, whether correctly or not (no behavioral EA). Other information collected included accuracy (i.e., total number of questions correctly answered), the point at which the participant quit the task (e.g., “During question 5”), and pre-task, midpoint (i.e., after question 5), and post-task perceived stress measured by ratings on the 0–100 Subjective Unit of Discomfort Scale (SUDS) (31).

### 2.4 Statistical analyses

The primary predictor, behavioral EA, was a dichotomous variable based on the AAT (1 = quitting the task prematurely, 0 = having answered all questions, correctly or not). The other predictor, self-reported EA, was a continuous variable assessed by the AAQ total score. The covariates (see below), including age, years of education, and psychological distress (BSI-18 Global Score), were all continuous variables. Accuracy and perceived stress (measured by the SUDS) in the behavioral EA task were used to assess behavioral EA task validity.

The primary outcome of the current study was the time-to-event variable, days until a participant either discontinued treatment before clinically recommended completion (event = 1) or was censored (event = 0), such that fewer days would indicate earlier treatment discontinuation.

Data analyses were performed using IBM SPSS Statistics (Version 29) (32). For descriptive statistics, continuous variables were summarized using mean and SD, and categorical variables were described using frequency and percentage. Bivariate analyses were conducted among participant characteristics, self-report measures, and AAT performance using Pearson’s correlations. To assess the validity of the behavioral EA task, paired *t*-test and fixed-effects analysis of covariance (ANCOVA) were used to examine perceived stress changes throughout the AAT in the whole sample and between individuals showing EA (i.e., avoiders) and those who did not (i.e., non-avoiders).

**Figure 2:**
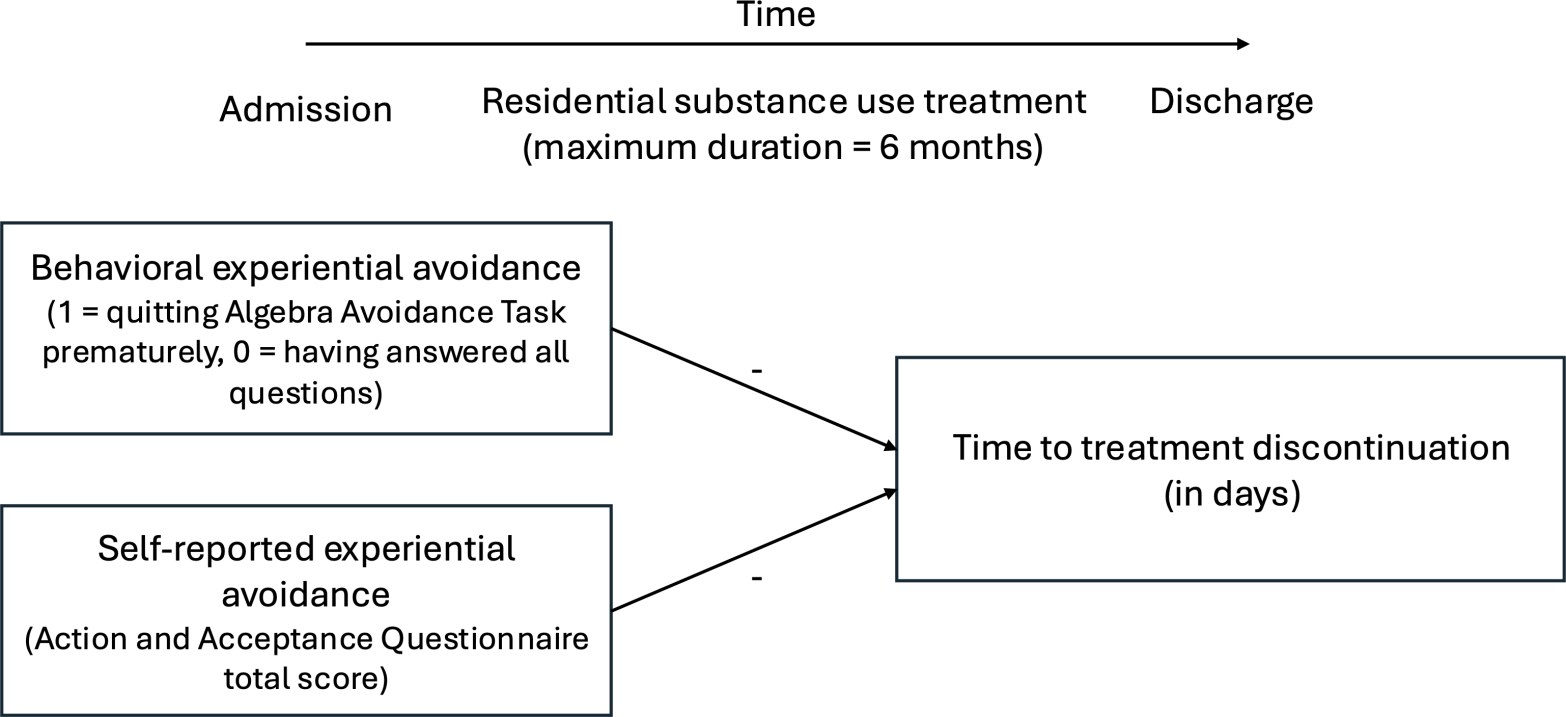
Schematic illustration of study variables and their associations.

The major analyses of the current study were Cox proportional hazards survival regressions on the association between behavioral/self-reported EA and time until treatment discontinuation. **Figure 2** presents a schematic illustration of the hypothesized associations. Two separate Cox regression models were constructed to examine the effect of behavioral and self-reported EA in predicting early treatment discontinuation. For each Cox regression model, age and years of education were entered as covariates as they were found by previous studies to be significant predictors of treatment completion status (5, 33). Psychological distress was also added as a covariate given its theoretical connections with both EA and treatment outcomes (19).

There was some evidence for the roles of prior treatment episodes and legal status in predicting SUD treatment outcomes in previous research (33, 34). In the current study, accuracy in the behavioral EA task was theoretically associated with the decision to quit the task early, as participants might be demotivated by poorer task performance. Therefore, sensitivity analyses were conducted to see if adding these variables as covariates would significantly change the results of the Cox regressions.

Potential multicollinearity among predictors was assessed by examining their correlations and the Variance Inflation Factor (VIF), with *r* < 0.80 and VIF< 10 evidencing absence of multicollinearity. A log-minus-log plot and the Schoenfeld residual tests were used to examine the proportional hazards assumption for each model. For the log-minus-log plot, parallel lines for the comparison groups would indicate proportional hazards assumption was fulfilled. For the Schoenfeld residual tests, a non-significant association between the partial residuals and time would validate the proportional hazards assumption. The Cox regression models’ fit was evaluated by examining the -2 Log Likelihood values and the omnibus test of model coefficients.

Exploratory analyses were conducted to explore other correlates of behavioral EA. Independent *t*-test was used to examine differences in age and years of education, and Chi-square test was used to examine differences in SUD subtype, legal status, and psychiatric medication between individuals demonstrating behavioral EA and those who did not. To account for the multiple comparison issue, Bonferroni correction was applied for these analyses with the significance level α = .05 being adjusted by the number of multiple comparisons (five in this case). A *p*-value less than or equal to the adjusted significance level $\alpha_{adjusted}$ = .01 was considered evidence for a significant association.

## 3 Results

### 3.1 Sample characteristics

Sixty of 97 clients who entered the residential treatment facility during the study period consented to participate. The first five participants were involved in an unpublished pilot study conducted to test study procedures, leaving 55 participants with formal data analyses. The pilot data suggested the self-report and behavioral measures being used were appropriate and effective.

**Table 1** summarizes the characteristics of 55 study participants at baseline. The participants had a mean age of 37.18 years (standard deviation [*SD*] = 10.90) and were all male. 90.9% participants were White. The mean years of education was 12.13 (*SD* = 1.59), with most participants having graduated from high school. 65.5% reported being single. 62.7% of participants were legally involved (i.e., receiving court-ordered evaluation, under probation, under parole). The most common primary SUD was alcohol use disorder (AUD; 43.6%). Most individuals reported multiple SUDs (83.6%). The mean number of prior residential substance use treatment episodes was 4.10 (*SD* = 3.17). 70.9% of participants were taking psychiatric medications with antidepressants being the most common medication.

**Table 1:** Participant characteristics at baseline (*n* = 55).

Characteristic	*n*	*%*
Age (*M, SD*)	37.18	10.90
Gender		
Male	55	100.0
Race/ethnicity		
Non-Hispanic White	48	87.3
Hispanic White	2	3.6
American Indian/Alaskan Native	3	5.5
Black/African American	1	1.8
Native Hawaiian/Other Pacific Islander	1	1.8
Years of education (*M, SD*)	12.13	1.59
Level of education		
Less than 9^th^ grade	2	3.6
9^th^ to 12^th^ grade, no diploma	6	10.9
High school graduate or higher	25	45.5
Some college or associate’s degree	22	40.0
Marital status		
Single	36	65.5
Married	2	3.6
Divorced	11	20.0
Separated	5	9.1
Widowed	1	1.8
Legal status at admission^a^		
Court-ordered presentence evaluation	2	3.7
Under parole	7	13.0
Under probation	27	50.0
Voluntary admission	18	33.3
Primary SUD diagnosis		
Alcohol	24	43.6
Cocaine/crack	1	1.8
Marijuana/hashish/pot	8	14.5
Other opiates and synthetics	5	9.1
Methamphetamine	17	30.9
Multiple SUD diagnoses	46	83.6
Number of prior residential substance use treatment episodes (*M, SD*)^b^	4.10	3.17
Taking psychiatric medication	39	70.9
Antidepressant	27	49.1
Antipsychotic	2	3.6
Anxiolytic	1	1.8
Multiple^c^	9	16.4
Receiving MAT^d^	3	5.6
BSI-18 (*M, SD*)	9.27	10.99
AAQ (*M, SD*)	33.84	3.95
DTS (*M, SD*)	3.77	0.90

AAQ, Acceptance and Action Questionnaire; BSI-18, Brief Symptom Inventory-18; DTS, Distress Tolerance Scale; M, mean; MAT, medication-assisted treatment; SD, standard deviation; SUD, substance use disorder.

a Reported by *n* = 54.

b Reported by *n* = 49.

c These could include any combinations among antidepressants, antipsychotics, mood stabilizers, anxiolytics, benzodiazepines, and stimulants.

d One participant reported nicotine transdermal patch, one reported naltrexone, and one reported acamprosate.

Of all 55 participants, twenty-six (47.3%) discontinued residential SUD treatment early and 29 (52.7%) completed it. Participants spent an average of 114.56 (*SD* = 92.33) days in treatment. They attended an average of 4.78 (*SD* = 2.98) 12-step meetings during treatment. Four (7.3%) participants returned to substance use during treatment. Reasons for treatment discontinuation included leaving against professional advice (i.e., leaving against professional recommendation to stay in residential treatment; *n* = 7), termination by the facility (*n* = 7), choosing to decline additional treatment (i.e., declining recommended additional outpatient treatment toward the end of residential stay; *n* = 6), incarceration (*n* = 3), and transfer to another facility (*n* = 3).

### 3.2 Descriptive statistics and bivariate analyses

Among the 55 participants, twenty-six (47.3%) quit the behavioral task early (i.e., EA) and 29 (52.7%) answered all nine test questions (i.e., no EA). The average number of questions answered correctly was 2.38 (*SD* = 1.33), indicating generally low accuracy. Notably, six (10.9%) participants were unable to answer any question correctly and no participant was able to correctly answer more than five out of the nine questions, suggesting that the task was indeed challenging for this sample. Detailed item-level performance is provided in **Supplementary Figures 1, 2**. A significant negative correlation was observed between test accuracy and behavioral EA, with lower accuracy being associated with a greater likelihood of EA (*r* = –.36, *p* = .007; **Supplementary Table 2**).

Regarding perceived stress, participants reported an average SUDS rating of 18.59 (*n* = 55, *SD* = 25.19) at the beginning of AAT, which increased to 45.96 (*n* = 46, *SD* = 30.48) at the midpoint, and 44.18 (*n* = 55, *SD* = 30.51) at the task’s conclusion. A paired *t*-test demonstrated a significant increase in perceived stress from the start to the midpoint, *t*(46) = 7.30, *p* <.001, and the end, *t*(54) = 6.93, *p* <.001, showing the task was indeed stress-inducing. ANCOVA was performed to examine differences in perceived stress changes between avoiders and non-avoiders, which revealed a significant main effect of time, *F*(1,53) = 48.25, *p* <.001, but no significant interaction between behavioral EA and time, *F*(1,53) = 0.69, *p* = .409, suggesting that perceived stress increased similarly for participants who demonstrated behavioral EA and those who did not. Neither did increase in perceived stress from the start to the midpoint of the task significantly differ between individuals who demonstrated behavioral EA and those who did not. These findings provided evidence for the stress induction effect of the AAT.

An examination of individual SUDS ratings (**Supplementary Figure 3**) revealed varied levels of perceived stress (ranges = 0–100) at the midpoint and the end of the task. Specifically, not every non-avoider experienced a high level of stress as they went through the questions. On the other hand, some avoiders experienced a relatively low level of stress. It seems that heightened stress was neither a sufficient nor necessary condition of the decision to quit the task. These results tentatively supported the validity of the AAT in assessing individual differences in the tendency to avoid distress regardless of the amount.

Zero-order correlations among study variables are presented in **Supplementary Table 2**. A shorter time to treatment discontinuation was significantly correlated with behavioral EA (*r* = .27, *p* = .05) and return to substance use during treatment (*r* = .30, *p* = .03). Unexpectedly, higher self-reported EA was significantly correlated with a lower likelihood of behavioral EA (*r* = –.31, *p* = .02), showing a negative association between self-reported and behavioral EA.

### 3.3 Survival analyses

Cox proportional hazards regressions were performed to assessing the effect of behavioral and self-reported EA in predicting early treatment discontinuation, controlling for age, years of education, and psychological distress. At baseline, there were only four missing values at the item-level among all AAQ (self-reported EA) and BSI-18 (psychological distress) items. Missingness was not significantly correlated with any participant characteristic, evidencing missing at random. The total scores were thus mean-imputed for outcomes with missing values. There were no concerns about multicollinearity (*r*’s<.40, VIFs< 2) of distinct predictors within the Cox models. For the Cox model using behavioral EA as the predictor, the proportional hazards assumption was met as evidenced by parallel lines in the log-minus-log plot (**Supplementary Figure 4**) and non-significant Schoenfeld residual tests (*p*’s >.05). For the Cox model using self-reported EA as the predictor, which was a continuous variable, a log-minus-log plot was not suitable as it would become “cluttered”. Therefore, only the Schoenfeld residual tests were conducted which yielded non-significant associations between the partial residuals and time (*p*’s >.05). Together, these results indicated no violations of the proportional hazards assumption.

**Figure 3:**
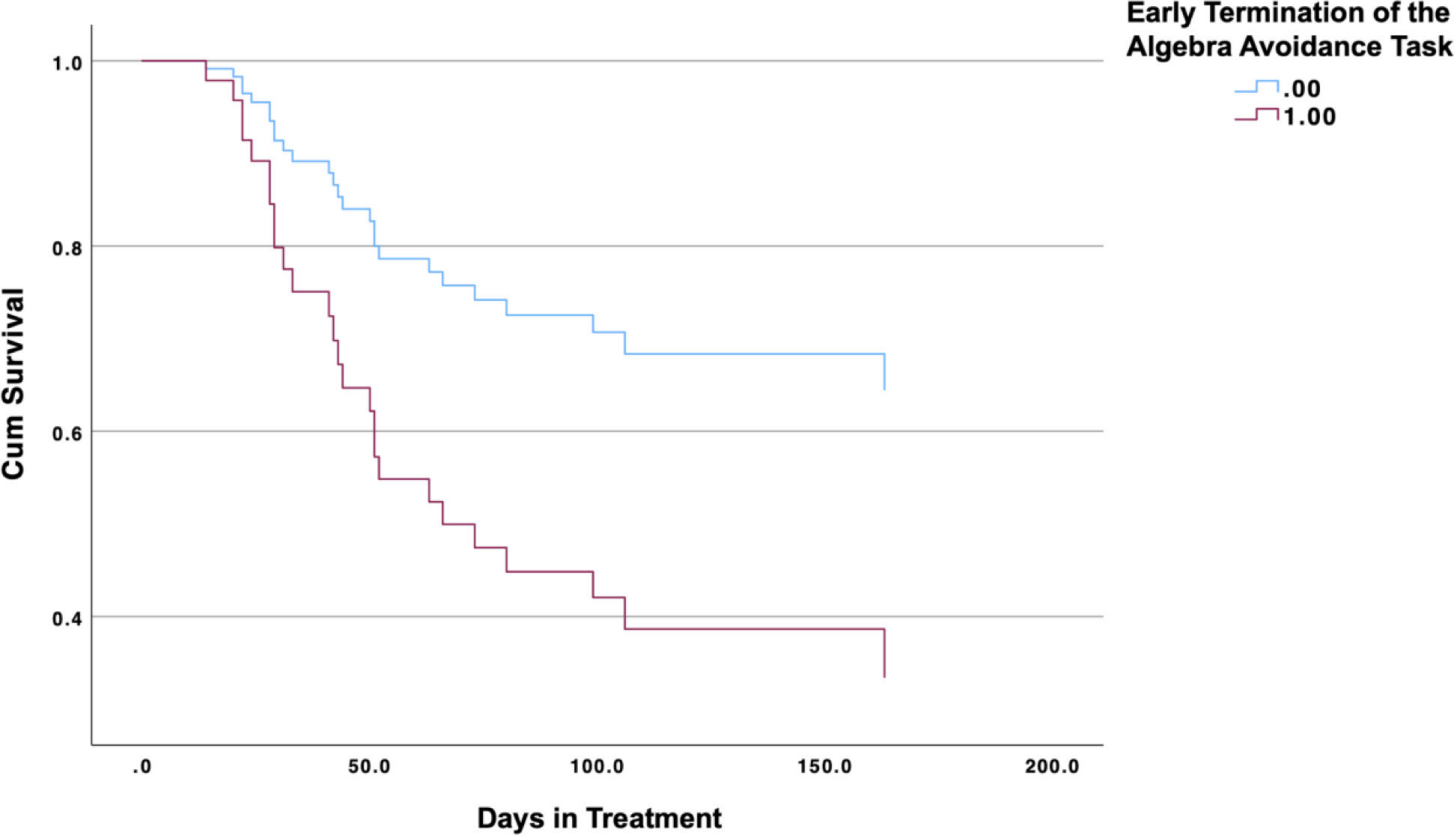
Survival plot of Cox hazards proportional regression analyses on behavioral experiential avoidance in predicting early discontinuation of substance use treatment.

**Table 2** summarizes the results of the Cox proportional hazards regression models. The model using behavioral EA as the predictor had a good fit, with a -2 Log Likelihood value of 177.27 and a significant omnibus test of model coefficients, *χ^2^*(4, *n* = 55) = 15.10, *p* = .004 (**Figure 3**). Behavioral EA, as indicated by quitting the behavioral EA task early, significantly predicted a shorter time to treatment discontinuation, *HR* = 2.50, 95% CI [1.09, 5.73], *p* = .030. Greater behavioral EA was thus significantly associated with an increased hazard of early treatment discontinuation.

**Table 2:** Results of the Cox proportional hazards regression analyses on behavioral and self-reported experiential avoidance in predicting early discontinuation of substance use treatment (*n* = 55).

Variable	*HR*	95% CI		*p*
		*LL*	*UL*	
Model 1: Behavioral EA				
Age	0.97	0.93	1.01	0.107
Years of education	0.99	0.76	1.29	0.948
BSI-18	1.05	1.02	1.09	0.001**
AAT termination	2.50	1.09	5.73	0.030*
Model 2: Self-reported EA				
Age	0.96	0.93	1.01	0.087
Years of education	0.93	0.72	1.21	0.599
BSI-18	1.05	1.02	1.09	<0.001***
AAQ total score	0.94	0.85	1.03	0.185

AAQ, Acceptance and Action Questionnaire; AAT, Algebra Avoidance Task; BSI-18, Brief Symptom Inventory-18; CI, confidence interval; EA, experiential avoidance; HR, hazard ratio; LL, lower limit; UL, higher limit.

*p<.05, **p<.01, ***p<.001

The Cox regression model using self-reported EA had a good fit, with a -2 Log Likelihood value of 180.48 and a significant omnibus test of model coefficients, *χ^2^*(4, *n* = 55) = 13.04, *p* = .011. However, self-reported EA, as measured by the AAQ total score, was not significantly associated with a shorter time to treatment discontinuation, *HR* = 0.94, 95% CI [0.85, 1.03], *p* = .185, showing that higher self-reported EA was not significantly linked to an increased hazard of early treatment discontinuation.

Finally, sensitivity analyses were conducted to see if the main Cox models held with number of previous residential treatment episodes, legal status, and behavioral EA task accuracy being added as covariates. Results showed that the addition of these covariates did not significantly alter the results (**Supplementary Table 3**), that is, behavioral EA remained to be a significant predictor of early treatment discontinuation, while self-reported EA remained non-significant, in the model that further accounted for previous residential treatment episodes, legal status, and behavioral EA task accuracy.

### 3.4 Exploratory analyses

Exploratory independent *t*-tests and Chi-square tests were used to examine differences between individuals who demonstrated behavioral EA and those who did not. The analyses revealed a significant group difference in SUD type: individuals with behavioral EA were more likely to have reported a primary AUD (62.1%) than a non-alcohol SUD (37.9%), χ^2^(1, *n* = 55) = 8.47, *p* = .006 (< $\alpha_{adjusted}$ = .01), relative to those without behavioral EA, showing that a primary AUD was significantly associated with a higher likelihood of behavioral EA. No other participant characteristic was found to significantly predict behavioral EA.

## 4 Discussion

This study examined EA, assessed both behaviorally and with a self-report measure, as a predictor of early discontinuation of residential substance use treatment among unhoused individuals. EA, the tendency to avoid distressing internal experiences, was linked to negative treatment outcomes (15, 17–19). As hypothesized, behavioral EA, assessed via tendency to prematurely end a stress-inducing algebra task, significantly predicted a shorter time to treatment discontinuation while accounting for psychological distress. Participants who demonstrated behavioral EA were more likely to leave treatment early, suggesting that avoidance directly hindered treatment engagement. Contrary to the hypothesis, self-reported EA did not predict early treatment discontinuation, emphasizing the utility of behavioral measures in capturing avoidance tendencies specific to treatment contexts.

The current findings contributed to the growing body of research linking EA to negative treatment outcomes, with implications for both clinical practice and theories of SUDs. For instance, Kohn et al. (2002) found that self-reported avoidant coping predicted shorter stays in outpatient treatment, controlling for mood symptoms (19). Our findings expanded this understanding by demonstrating the predictive value of behavioral EA in residential settings. The current results aligned with the punishment-avoidant learning model (35) by showing that individuals reduce behaviors associated with short-term negative outcomes (e.g., distress) despite their potential longer-term benefits. EA may limit an individual’s ability to endure short-term discomfort in favor of longer-term goals, leading to treatment discontinuation. This mechanism may be explained by the negative reinforcement hypothesis, which posits that substance use behaviors are maintained by temporary relief from negative emotions (36). Interestingly, during the current algebra task, both avoiders and non-avoiders reported increased stress upon the end of task, implying that quitting the challenging mathematics task may not have led to immediate stress relief.

A key finding of this study is the ability of behavioral EA to predict treatment discontinuation above and beyond the effect of global psychological distress (37). AAT thus provided a task-based measure of EA that captured behavioral tendencies influencing treatment engagement, replicating findings on DT using stress-inducing tasks (19–22). Some may argue that our index of EA, the decision to quit the behavioral task, is conceptually related to lack of persistence through the task and has been used to measure DT (38, 39). Here, we propose that the decision to quit and lack of persistence do not necessarily capture the same construct. An individual may quit the task because i) the distress has exceeded the amount that they can withstand (i.e., DT), or ii) they tend to cope by avoiding stressful situations regardless of stress level (i.e., EA). We interpreted the decision to quit as EA rather than DT because it directly reflected the behavioral tendency of avoidance. Consistent with our interpretation, participants were found to quit the task while going through varied levels of stress, demonstrating a dissociation between the amount of distress being endured and the decision to quit.

To explore the role of DT in predicting early treatment discontinuation in the current sample, an additional Cox regression analysis was conducted using DTS total score as the predictor (see results in **Supplementary Table 4**). Results showed that unlike behavioral EA, DT did not significantly predict early treatment discontinuation. Previous research found a non-significant association between self-reported and behavioral indices of DT, implying that they may have captured distinct components of DT (40). Our findings were consistent with this literature and underscored the need to include both self-reported and behavioral assessments of emotion regulation processes.

The current study revealed a non-significant association between self-reported EA and treatment retention. Furthermore, self-reported EA was negatively correlated with behavioral EA. Pending validation from further research, AAQ may assess the emotional and cognitive (e.g., “Anxiety is bad”) more than the behavioral dimensions of avoidance (10). This divergence again highlighted the importance of using task-based measures in the context of SUD treatment-related behaviors.

An unexpected finding was that individuals with a primary AUD were more likely to demonstrate low EA compared to those with other primary SUDs. There is some evidence that alcohol and other substance use had differential associations with punishment and reward sensitivity (41). However, caution is needed in interpreting the result given its exploratory nature.

The theoretical relevance of this study lies in its integration of behavioral theories, such as negative reinforcement and positive punishment, to explain substance use treatment engagement. Compared to the negative reinforcement account, positive punishment theory posits that individuals with EA may leave treatment to avoid associated challenges, despite potential long-term benefits (15). The current behavioral EA task required participants to balance potential rewards (monetary incentives) against the stress of solving challenging problems, simulating a multigoal learning task. EA may impair an individual’s flexibility in learning from both reinforcement and punishment, prioritizing immediate stress avoidance over long-term gains. This inflexibility was linked to premature discontinuation of residential treatment (42). Understanding these mechanisms could inform the development of interventions targeting avoidance behaviors to improve treatment retention.

Clinically, the current results suggested that behavioral measures, like the AAT, could serve as valuable tools for identifying individuals at risk of early treatment discontinuation. Behavioral tasks provide actionable insights into real-world avoidance tendencies, enabling targeted interventions to address these behaviors (e.g., mindfulness, psychoeducation, attentional bias modification) (43). Differences in EA by SUD type further highlighted the need for tailored approaches, particularly for individuals with heightened avoidance tendencies or unique punishment-reward profiles.

This study has several strengths, including the use of both behavioral and self-reported measures of EA, enhancing the assessment of a multifaceted construct. Conducting the study in an active treatment facility bolstered its ecological validity and supported the translation of findings into clinical practice. The use of Cox regression survival analysis enabled modeling of discontinuation risk while accounting for multiple covariates, offering insights into the mechanisms driving treatment disengagement.

However, the following limitations tone our interpretation. First, due to data collection from a single treatment facility, the current study has a relatively small sample (*n* = 55) which may have limited the power to detect statistical significance. Second, the sample lacked diversity in gender, race/ethnicity, and geographic location. The male-only sample, while providing a homogeneous group for analysis, might not represent broader treatment populations. Third, some clinical data were not available to the research team, such as the residents’ smoking status, which could be a “marker of risk” in SUDs. Neither were we able to obtain a detailed treatment history (e.g., non-residential treatments received, duration of abstinence prior to the intake) other than the number of previous residential substance use treatment episodes. Future studies should examine these variables for a more thorough assessment of participants’ risk for negative treatment outcomes. Fourth, the AAT was a newly developed task pending validation in residential SUD treatment settings, which warranted caution in interpreting our findings. That said, examinations of perceived stress ratings throughout the task evidenced its validity in this sample. The use of a mathematics-based task may also have introduced confounds related to participants’ educational experiences, though *post-hoc* analyses found no significant correlation between years of education and distress changes during the task.

Another limitation is that participants left treatment early for a variety of reasons which might not be similarly driven by EA. Due to our small sample size, we were unable to compare the effects of treatment-related (e.g., leaving against professional advice) and non-treatment-related (e.g., termination by facility) reasons in predicting early treatment discontinuation. Future research should extend our findings by assessing the outcomes of residents who terminate treatment early for distinct reasons. Sixth, this study focused on early treatment discontinuation and return to use, excluding other relevant treatment outcomes (e.g., changes in substance use severity, psychosocial functioning). Lastly, it should be noted that the current data was collected over a decade ago (January to October 2014), which limited the generalizability of the present findings as policies and clinical practices in SUD treatment might have changed over the past decade.

## 5 Conclusions

In conclusion, this study underscored the significant role of behavioral EA in predicting early treatment discontinuation among unhoused individuals with severe SUDs. Unlike self-report measures, behavioral tasks like the AAT provided a proximal and contextually relevant assessment of avoidance tendencies, directly tied to treatment engagement. The current findings emphasized the importance of addressing EA in clinical interventions to improve treatment retention. Future research should explore the pathways linking EA to treatment outcomes, particularly the roles of negative reinforcement, positive punishment, and multigoal learning in shaping substance use recovery behaviors.

## Data Availability

The raw data supporting the conclusions of this article will be made available by the authors, without undue reservation.
