# Supplementary material for: Behavioral, not self-reported, experiential avoidance predicts early treatment discontinuation at residential substance use treatment
Source: Front Psychiatry. 2026 Feb 6;17:1725326. doi: 10.3389/fpsyt.2026.1725326 (PMC12920210; doi:10.3389/fpsyt.2026.1725326)
Supplement: Supplementary file 1 [file Table1.docx]

Supplementary Material

# Supplementary Material 1. Questions in the Algebra Avoidance Task.

**Practice Questions**

1. John bought 6 apples for 25 cents each. What was the total cost of the 6 apples (in dollars)? (Answer: 1.50)
2. John cut 6 apples into quarters. He divided the pieces of apple evenly among 8 friends. How many pieces of apple did each friend get? (Answer: 3)

**Test Questions**

1. Bill drove from Boston to Cleveland, a distance of 627 miles, in 11 hours. How fast did he drive (in miles per hour)? (Answer: 57)
2. A man's grocery bill is $8, but the store deducts $2 from his bill for coupons. If the man gives the grocery clerk $10, how much change should he get (in dollars)? (Answer: 4)
3. The members of a club decided to wash cars in order to earn money for the club. Each member of the club washed 3 cars and charged $2 per car. At the end of the day, the club had $66, which included $6 in tips. How many people were in the club? (Answer: 10)
4. Tammy has $9.70 in nickels, dimes, and quarters. The number of nickels is 4 more than 3 times the number of dimes, and the number of quarters is 5 fewer than 2 times the number of nickels. How many nickels does Tammy have? (Answer: 19)
5. The Elixir of Life consists of a total of 12 liters containing two solutions: Magic and Triple E. Magic is composed of three solutions: E, Double E, and Triple E in the ratio of 1:2:3, respectively. The concentration of the Elixir’s secret ingredient in E is 2%; Double E contains 2.5 times as much secret ingredient as E; and Triple E contains twice as much secret ingredient as Double E. The concentration of secret ingredient in the Elixir itself is 8%. How much Triple E is contained in the Elixir (in liters)? (Answer: 8)
6. Seiji invested some money and in three years earned a total of $100 less than 750% of his original investment. The first year, his earnings were $190 less than 300% of his original investment. The second year, he earned $340 more than 50% of his first-year earnings. The third year, he earned $314 less than 150% of his second-year earnings. How much money did Seiji originally invest (in dollars)? (Answer: 278)
7. Two sisters, Alice and Beth, left their house at the same time and rode their bicycles in opposite directions along a straight road. Alice rode at 4 mph, while Beth rode at 8 mph. In how many hours will they be 36 miles apart? (Answer: 3)
8. A florist has a total of 198 roses divided into bunches of a dozen or a half-dozen. There are 15 more bunches of a half-dozen than bunches of a dozen. How many bunches of a dozen are there? (Answer: 6)
9. Liz is 1 year younger than 3 times her sister Amanda’s age. Brother Roger is half as old as Liz will be 5 years from now. Their mother is 5 years younger than 7 times Roger’s age two years ago. Their father is 2 years older than their mother. Four years from now, the sum of the three children’s ages will be 14 more than one-fourth the sum of their parents’ ages at that same time. How old is Liz now? (Answer: 11)

**Supplementary Table 1**

Time allotments in the Algebra Avoidance Task.

| **Question no** | 1 | 2 | 3 | 4 | 5 | 6 | 7 | 8 | 9 |
| --- | --- | --- | --- | --- | --- | --- | --- | --- | --- |
| **Time allowed (in minute)** | 1 | 1 | 2 | 2 | 2 | 3 | 3 | 3 | 3 |

Total time allowed: 20 minutes.

**Supplementary Figure 1.** Number of participants who correctly answered certain number of questions in the algebra avoidance task (*n* = 55).

**
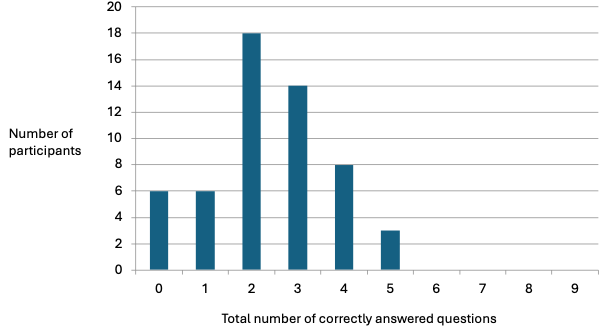
**

**Supplementary Figure 2.** Number of participants who attempted to answer and who correctly answered each question of the Algebra Avoidance Task (*n* = 55).

**Supplementary Table 2**

Zero-order correlations among self-report measures.

|  | Variable | 1 | 2 | 3 | 4 | 5 | 6 | 7 | 8 | 9 | 10 | 11 | 12 | 13 |  |
| --- | --- | --- | --- | --- | --- | --- | --- | --- | --- | --- | --- | --- | --- | --- | --- |
| 1 | Age |  |  |  |  |  |  |  |  |  |  |  |  |  |  |
| 2 | Years of education | –.09 |  |  |  |  |  |  |  |  |  |  |  |  |  |
| 3 | Number of prior SU treatment episodes^a^ | –.12 | –.13 |  |  |  |  |  |  |  |  |  |  |  |  |
| 4 | Taking psychiatric medication(s) | –.14 | .38** | –.13 |  |  |  |  |  |  |  |  |  |  |  |
| 5 | Days in treatment | .13 | .002 | –.13 | .19 |  |  |  |  |  |  |  |  |  |  |
| 6 | Early treatment discontinuation | –.17 | –.03 | .13 | –.16 | –.67** |  |  |  |  |  |  |  |  |  |
| 7 | Number of 12-step meetings attended^b^ | .10 | .05 | .10 | –.13 | .12 | –.15 |  |  |  |  |  |  |  |  |
| 8 | Return to substance use during treatment | –.04 | .11 | –.20 | .19 | –.14 | .30* | –.20 |  |  |  |  |  |  |  |
| 9 | Behavioral EA | .05 | –.24 | –.002 | –.16 | –.09 | .27* | –.26 | .02 |  |  |  |  |  |  |
| 10 | AAT accuracy | –.15 | .35** | –.09 | .16 | .003 | –.03 | .16 | –.03 | –.36** |  |  |  |  |  |
| 11 | BSI-18 | –.26 | .18 | –.16 | .02 | .17 | –.17 | .001 | –.03 | –.05 | .05 |  |  |  |  |
| 12 | AAQ | –.20 | .10 | –.17 | .10 | –.11 | –.10 | –.05 | .03 | –.31* | .19 | .01 |  |  |  |
| 13 | DTS | –.09 | –.04 | .05 | –.12 | –.06 | –.07 | –.09 | –.20 | –.12 | .14 | –.23 | .08 |  |  |
| Abbreviations: AAQ = Acceptance and Action Questionnaire; AAT = Algebra Avoidance Task; BSI-18 = Brief Symptom Inventory-18; DTS = Distress Tolerance Scale; SU = substance use.  ^a^Reported by *n* = 49.  ^b^Reported by *n* = 53.  **p* < .05, ***p* < .01, ****p* < .001 | | | | | | | | | | | | | | | |

**Supplementary Figure 3.** Individual ratings on the Subjective Unit of Discomfort Scale at the beginning, midpoint, and end of the Algebra Avoidance Task for participants with behavioral experiential avoidance (upper) and those without (lower).

**
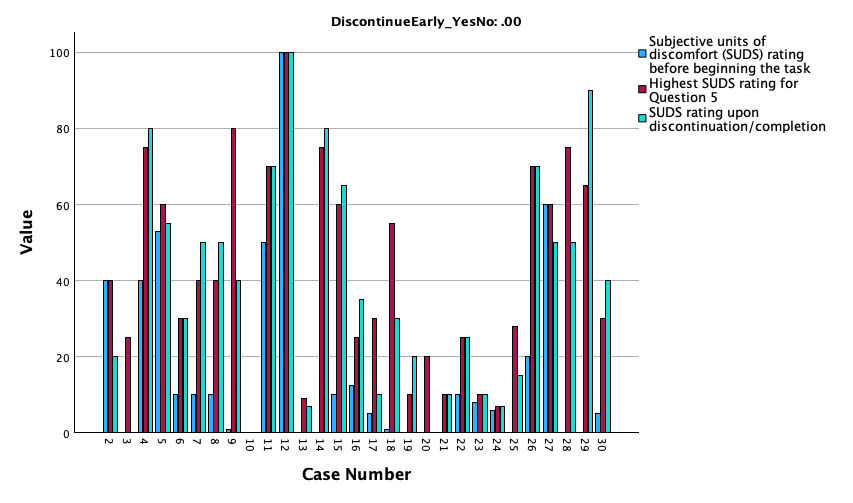
**

**
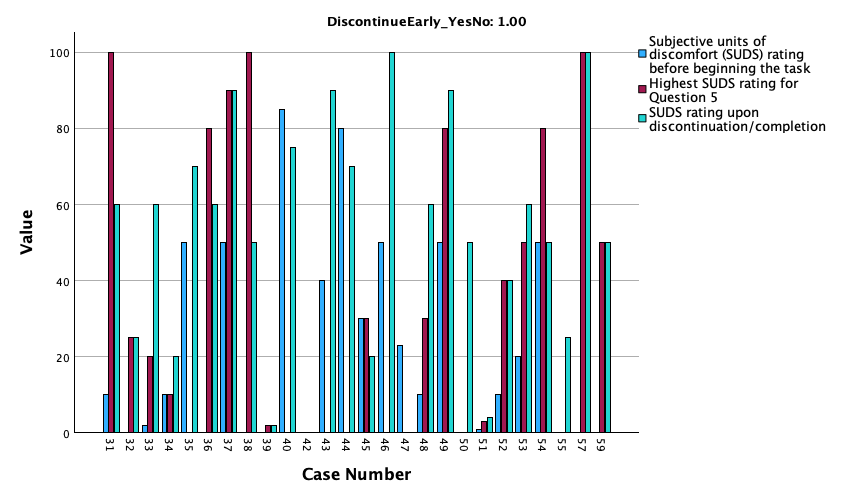
**

**Supplementary Figure 4.** Log minus log plot for Cox proportional hazards regression assessing the effect of behavioral experiential avoidance in predicting early treatment discontinuation.

**Supplementary Table 3**

Sensitivity analyses on Cox proportional hazards regression analyses on behavioral experiential avoidance with number of previous substance use treatment episodes, legal status, and Algebra Avoidance Task accuracy included as covariates (*n* = 48).

| Variable | *HR* | 95% CI | | *p* |
| --- | --- | --- | --- | --- |
|  |  | *LL* | *UL* |  |
| **Model 1: Behavioral EA** |  |  |  |  |
| Age | 0.97 | 0.93 | 1.01 | 0.128 |
| Years of education | 1.05 | 0.76 | 1.45 | 0.781 |
| BSI-18 | 1.04 | 1.01 | 1.08 | 0.020* |
| Number of prior residential substance use treatment episodes^a^ | 1.04 | 0.92 | 1.17 | 0.572 |
| Legal status at admission^b^ | 1.29 | 0.50 | 3.29 | 0.601 |
| AAT accuracy | 1.00 | 0.69 | 1.45 | 0.988 |
| AAT termination | 2.72 | 1.00 | 7.37 | 0.049* |
| **Model 2: Self-reported EA** |  |  |  |  |
| Age | 0.95 | 0.91 | 1.00 | 0.05* |
| Years of education | 0.98 | 0.71 | 1.36 | 0.91 |
| BSI-18 | 1.05 | 1.02 | 1.09 | 0.006* |
| Number of prior SU treatment episodes^a^ | 0.96 | 0.85 | 1.10 | 0.58 |
| Legal status^b^ | 0.86 | 0.31 | 2.36 | 0.770 |
| AAT accuracy | 0.89 | 0.64 | 1.24 | 0.491 |
| AAQ total score | 0.89 | 0.78 | 1.01 | 0.072 |
| Abbreviations: AAQ = Avoidance and Acceptance Questionnaire; AAT = Algebra Avoidance Task; BSI-18 = Brief Symptom Inventory-18; EA = experiential avoidance; *HR* = hazard ratio; *LL* = lower limit; *UL* = higher limit; SU = substance use.  ^a^Reported by *n* = 49.  ^b^Reported by *n* = 54.  **p* < .05, ***p* < .01, ****p* < .001 | | | | |

**Supplementary Table 4**

Results of the Cox proportional hazards regression analyses on self-reported distress tolerance in predicting early discontinuation of substance use treatment (*n* = 55).

| Variable | *HR* | 95% CI | | *p* |
| --- | --- | --- | --- | --- |
|  |  | *LL* | *UL* |  |
| Age | 0.97 | 0.94 | 1.01 | 0.142 |
| Years of education | 0.92 | 0.73 | 1.18 | 0.523 |
| BSI-18 | 1.05 | 1.02 | 1.08 | 0.003** |
| DTS total score | 1.00 | 0.60 | 1.66 | 0.998 |
| Abbreviations: BSI-18 = Brief Symptom Inventory-18; DTS = Distress Tolerance Scale; *HR* = hazard ratio; *LL* = lower limit; *UL* = higher limit.  **p* < .05, ***p* < .01, ****p* < .001 | | | | |
